# Vienna Letter

**Published:** 1881-06

**Authors:** Wm. T. Belfield

**Affiliations:** Vienna


### Article VII.

Vienna, March 26, 1881.

Editors Chicago Medical Journal and Examiner:—I sent you last month a description of the first successful resection of the stomach for cancer of the pylorus, performed Jan. 29, by Billroth. At that time the recovery of the patient, though probable, was by no means assured. The subsequent history of the case, however, has justified the most sanguine anticipations—not a single unfavorable symptom occurred; the patient was in two weeks discharged recovered, and has since remained quite well.

Encouraged by this success Billroth repeated the operation February 28, on a very anæmic woman, 39 years old, who had suffered from gastric derangement for seven months, and had presented the usual symptoms of commencing cancer of the pylorus during the previous seven weeks. In this case two difficulties were encountered which rendered the operation decidedly more formidable—first, a dilatation of the stomach, whereby the preliminary cleansing of the organ was rendered impossible; second, an adhesion of the stomach to the parietal peritoneum (as revealed by the exploratory incision) in consequence of ulceration and adhesive peritonitis. The operation was in general upon the same plan as the first, yet complicated by the sponging out of the gastric contents, and by the removal of the cancerous infiltration in the abdominal wall at the seat of ulceration from the stomach. The portion of stomach excised was 10 ctm. long and 6 wide—a medullary cancer. Fifty-eight stitches were made in the stomach and duodenum, the operation lasting two and three quarters hours. During the first day after the operation, ice only was administered per orem, sour milk and ice the second day; nourishing clysters were given during the entire period. The patient vomited once on the second day, but on the third and succeeding days constantly. The stomach could be induced to retain nothing whatever more than three or four hours; the vomit contained acid, gastric juice and bile. As this condition of things, which Billroth ascribed, 1st, to a mechanical obstacle (knicking) to the passage of contents from the dilated stomach into the duodenum; and, 2d, to imperfect contractions of the stomach in consequence of dilatation and of adhesions to the abdominal wall at point of excision of latter, threatened to destroy the patient by inanition, Billroth decided to reopen the wound, a decision which he carried into execution on the seventh day after the operation. His fears were realized—a very sharp angle existed at the line of junction of the duodenum with the enormously dilated stomach, and adhesion existed with the diaphragm above as well as with the abdominal wall in front. Billroth sewed the edges of the stomach to the abdominal wall, and introduced a drainage-tube as large as the finger into the abdomen for purposes of nutrition. The patient died the next (8th) day of inanition. The section proved that no peritonitis had at any time existed.

On the 12th of March Billroth performed the third resection of the stomach, this time on a 36-year old cachectic woman. In order to avoid the danger of an angle at the point of junction, he sewed the duodenum to the stomach at a point low down on the greater curvature, hoping thus to facilitate the exit of the gastric contents. The value of this measure was not, however, ascertained, since the patient died twelve hours later of shock.

Although the present percentage is not very encouraging, Billroth is evidently determined to test the operation further, believing that as he learns the various modifications required in the operation by the various conditions of the organs concerned, a greater degree of success can be expected. After his return from his present vacation (about May 1) we shall probably hear of further cases. WM. T. BELFIELD.

